# Ecophysiological Leaf Traits of Forty-Seven Woody Species under Long-Term Acclimation in a Botanical Garden

**DOI:** 10.3390/plants11060725

**Published:** 2022-03-09

**Authors:** Qinglin Sun, Liming Lai, Jihua Zhou, Xin Liu, Yuanrun Zheng

**Affiliations:** 1 Key Laboratory of Resource Plants, West China Subalpine Botanical Garden, Institute of Botany, Chinese Academy of Sciences, Beijing 100093, China; sunqinglin@ibcas.ac.cn (Q.S.); lailiming@ibcas.ac.cn (L.L.); zhoujihua@ibcas.ac.cn (J.Z.); liuxin@ibcas.ac.cn (X.L.); 2 University of Chinese Academy of Sciences, Beijing 100049, China

**Keywords:** ecophysiological traits, green plants, long-term acclimations, botanical garden, life form

## Abstract

Ex situ conservation plays an important role in the conservation and utilization of plant resources. In recent years, botanical gardens have greatly improved the ex situ conservation of plants, and research has mainly focused on morphological characteristics, reproduction technology, and conservation value. There are few studies on the ecophysiological traits of plants after conservation. Forty-seven plants that are frequently used in North China and were grown in the Beijing Botanic Garden were selected to measure their photosynthetic traits, light-use efficiency (LUE), water–use efficiency (WUE), specific leaf area (SLA), relative chlorophyll content (SPAD), and leaf water potential (φ). An analysis of variance showed that there were significant differences in the ecophysiological traits of the leaves of 47 woody species. The light saturation point (LSP), net photosynthetic rate at light saturation (Pnmax), φ, and SLA had significant differences among different plant life forms. The SLA and SPAD of leaves were significantly different among the families. The LUE of all species reached its maximum under a low light intensity, and species with a large difference between the light saturation point and light compensation point had larger Pnmax values. This research further adds to the understanding of the adaptation mechanisms of plants to the environment under the conditions of a botanical garden as well as the environmental fitness in a long-term ex situ domestication and then helps with scientifically setting up artificial management conditions.

## 1. Introduction

In recent years, affected by global climate change and an intensification of human activities, many plants have been in a state of endangered extinction, leading to a loss of biodiversity [1]. The conservation and utilization of plant resources is currently one of the most important development strategies in China and even around the world [2]. Woody plants are of immense economic, cultural, and ecological importance, and efforts are urgently required to prevent the loss of woody species and the associated ecosystem services that they support [3]. As an important measure to save plant species, ex situ conservation is an important part of biodiversity protection and is also the basis for the return of rare and endangered plants to their natural habitats, the restoration and reconstruction of wild populations, and the guaranteeing of original materials, which play pivotal roles in conservation and utilization [4,5].

Botanical gardens are an important part of ex situ conservation, are important for the protection of living plants, and they play important roles in environmental beautification, cultural leisure, and climate regulation [6]. The plants grown in botanical gardens are different from those in the wild environment and are usually under artificial management and conservation conditions; the grounds are often carefully mulched, weeded, fertilized, water irrigated, and free of pests. Individual plants are often well spaced and grown under conditions that are considered ideal for their species, which affect their ecophysiological traits [7,8]. In recent years, various botanical gardens have performed significant work in the ex situ conservation of plants and have reserved a large amount of plant resources [9]. This research mainly focuses on morphological characteristics, reproduction technology, protection value, etc., and has solved the problem of the reproduction and cultivation of a group of rare and endangered plants [10]. However, the domestication and utilization of resources is still lagging somewhat behind. The function of botanical gardens is not closely integrated with scientific research, and the resource allocations of a botanical garden are unreasonable, which leads to the phenomenon of poor growth and reproductive failure of plants growing in a conservation environment for a long time [10,11]. This restricts the effective protection and sustainable utilization of plants with high economic value or that are rare and endangered [9].

Different types of garden plants have different ecophysiological traits, and different plants have different adaptabilities to the external environment and their own internal adjustment capabilities [12,13]. The ecophysiological traits of plant leaves have been shown to vary according to environmental conditions; through adaptation and acclimation, environmental factors have a strong influence over ecophysiological traits, which can reflect the growth and resistance of plants to a certain extent [14,15]. Photosynthesis is the basis of plant growth and development, and photosynthetic traits are the most important ecophysiological traits of plants [16,17]. The photosynthetic physiological traits of plant leaves reflect plant growth strategies and resource utilization; plants interact with the environment for a long time, and are constantly adapting to the external environment [18]. Light and water are the most important environmental factors for plant growth and development. These are the power and important energy sources for photosynthesis, and are also important factors that limit the distribution of plants in nature and affect plant productivity [12]. There have been many studies on the ecophysiological traits of species, with most of them mainly being studies on the relationship between plant leaf ecophysiological traits and environmental factors from the perspective of control and pot experiments [13]. However, there are few studies on the analysis of ecophysiological plant traits in different life forms, families, and different species in the same or similar environments. Plant life forms are a combination of a series of plants with certain functional characteristics, which can be regarded as a combination that has the same response to an environment and similar effects in terms of the main ecosystem processes [19,20]. Different plant life forms have different plant functional traits, thus maximizing the utilization of resources and reducing adverse environmental impacts [20]. The ecophysiological traits of plant life forms depend on the ecophysiological traits of different plants in the life form [20]. Studies have shown that trees and shrubs have different ecophysiological traits at different levels [15]. In botanical gardens, the planting density of species is sparse, and there is less of a multi-layer structure. As they are affected by artificial watering and irrigation, plants in a botanical garden are exposed to environmental conditions of having sufficient light and water over a long period of time [7,8]. Evaluating the ecophysiological traits of woody species conserved in similar environments is useful for us to understand plant adaptation and improve plant conservation. 

Taking a garden as the example to study the ecophysiological traits of species under artificial planting and conservation conditions, and based on parameters from the light-response curve, light-energy and water-use efficiency, physiological water traits, specific leaf area (SLA), and relative chlorophyll content (SPAD), we aimed to answer the following questions: (a) what are the differences in the ecophysiological indicators of different woody species in the same environment; and (b) how did ecophysiological traits change across different plant life forms and families? 

## 2. Results

### 2.1. Light-Response Curves of Different Species

**Figure 1:**
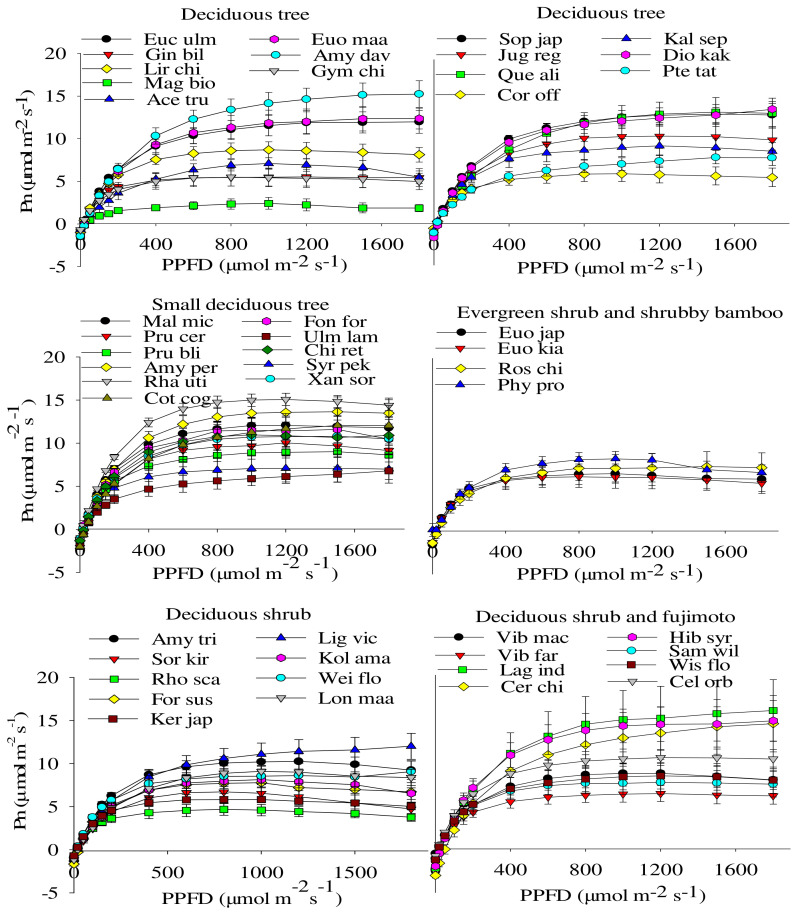
Light photosynthetic response curves (mean ± SE, *n* = 5) of forty-seven woody species. Pn and PPFD are net photosynthetic rate and photosynthetic photon flux density, respectively. Species abbreviations are shown in Table 1.

The responses of the net photosynthetic rate (Pn) of 47 species to photosynthetic photon flux densities (PPFD) were basically similar and could generally be divided into three stages: straight-line increase, curved increase, and steady (Figure 1). The curves of a few tree species showed a downward trend after reaching stability. The straight up phase was under weak light conditions (<200 μmol·m^−2^·s^−1^), the curve increased from weak light to saturated light intensity, and the photosynthetic rate increased with increasing light intensity. Pn was limited by various factors within this range. In the saturation stage after reaching saturated light intensity, Pn no longer increased with increasing PPFD. A few species showed a downward trend, such as *Acer truncatum* Bunge, *Fontanesia fortunei* Carrière, *Phyllostachys propinqua* McClure, *Sorbaria kirilowii* Maxim, *Kolkwitzia amabilis* Graebn., *Amygdalus triloba* Ricker, *Lonicera maackii* Maxim., and *Viburnum macrocephalum* Fort. f. *keteleeri* (Carrière) Rehder. Deciduous trees, small deciduous trees, and deciduous shrubs and fujimoto had large amplitude changes in their light-response curves, whereas evergreen shrubs and shrubby bamboo had small amplitude changes. *Amygdalus davidiana* Carrière had the highest net photosynthetic rate, and *Magnolia biondii* Pamp. had the lowest net photosynthetic rate among deciduous trees. *Rhamnus utilis* Decne. had the highest net photosynthetic rate, and *Ulmus lamellosa* Wang had the lowest net photosynthetic rate among small deciduous trees. *Lagerstroemia indica* L. had the highest net photosynthetic rate, and *Rhodotypos scandens* Makino had the lowest net photosynthetic rate among the deciduous shrubs and fujimoto (Figure 1).

### 2.2. Water-Use Efficiency (WUE) and Light-Utilization Efficiency (LUE) Curves of Different Species

**Figure 2:**
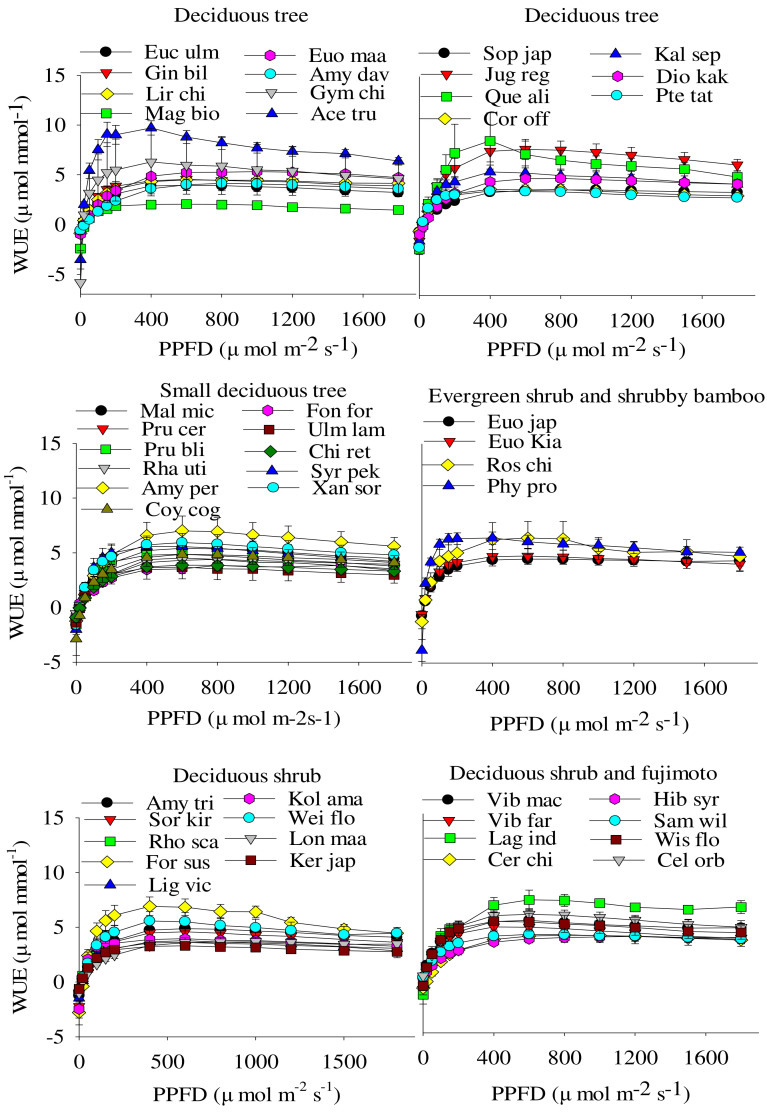
Water-use efficiency curves (mean ± SE, *n* = 5) of forty-seven woody species. WUE and PPFD are water-use efficiency and photosynthetic photon flux density, respectively. Species abbreviations are shown in Table 1.

Similar to the responses of Pn to PPFD, the WUE responses of 47 woody species to PPFD were basically similar and could generally be divided into three stages: straight-line increase, curved increase and steady (Figure 2). The curves of a few woody species showed a downward trend after reaching stability, such as *A. truncatum*, *Gymnocladus chinensis* Baill, *Quercus aliena* Bl. var. *Acuteserrata* Maxim., *P. propinqua* and *Forsythia suspensa* Vahl. *A. truncatum* had the highest WUE, and *M. biondii* had the lowest WUE among the deciduous trees. *Amygdalus persica* L. had the highest WUE, and *U. lamellosa* had the lowest WUE among the small deciduous trees. *P*. *propinqua* had the highest WUE, and *Euonymus japonicus* Thunb. had the lowest WUE among the evergreen shrubs and shrubby bamboo. *L. indica* had the highest WUE, and *Kerria japonica* L. had the lowest WUE among the deciduous shrubs and fujimoto. The WUE of most woody species was stable at around 5 μmol·mmol^−1^ (Figure 2). 

**Figure 3:**
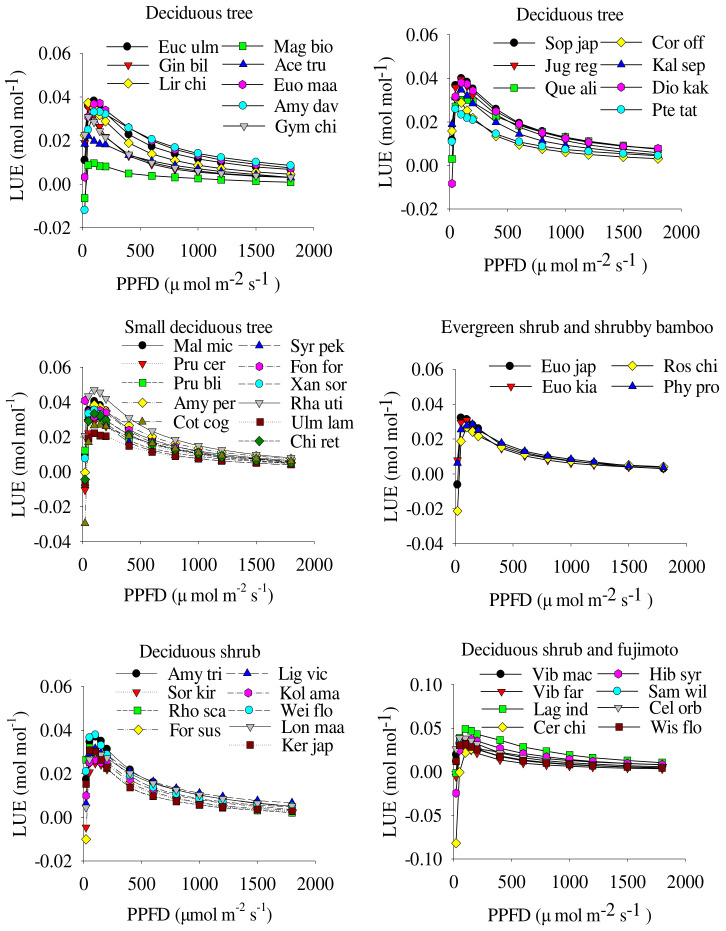
Light-energy-utilization efficiency curves (mean ± SE, *n* = 5) of forty-seven species. LUE and PPFD are light-utilization efficiency and photosynthetic photon flux density, respectively. Species abbreviations are shown in Table 1.

The LUE of species leaves increased rapidly with the increase in PPFD, and after reaching the maximum value, it decreased non-linearly with the increase in PPFD. LUE reached its maximum under low PPFD, and the LUE of woody species were similar under a high PPFD (Figure 3). Deciduous trees, small deciduous trees and deciduous shrubs and fujimoto had large amplitude changes in their LUE curves, whereas evergreen shrubs and shrubby bamboo had small amplitude changes. *Sophora japonica* L. had the highest LUE, and *M. biondii* had the lowest LUE among the deciduous trees. *R. utilis* had the highest LUE, and *U. lamellosa* had the lowest LUE among the small deciduous trees. *E. japonicus* had the highest LUE, and *Rosa chinensis* Jacq. had the lowest LUE among the evergreen shrubs and shrubby bamboo. *L. indica* had the highest LUE, and *K. japonica* had the lowest LUE among the deciduous shrubs and fujimoto (Figure 3). 

### 2.3. Ecophysiological Leaf Traits of 47 Species

**Figure 4:**
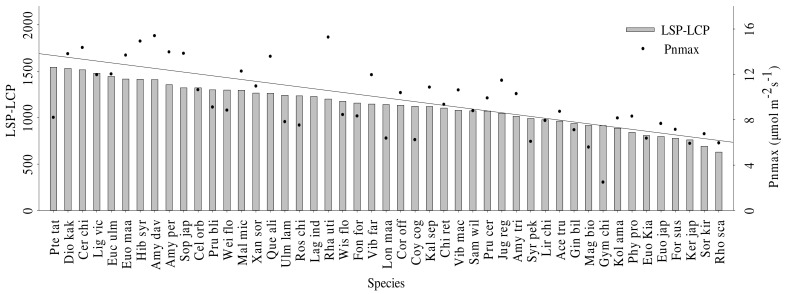
The relationship between light intensity range and Pnmax of different species. Each bar represents the mean of five replicates. LSP, LCP and Pnmax are light saturation point, light compensation point, and net photosynthetic rate at light saturation, respectively. Species abbreviations are shown in Table 1.

The F-values of the one-way analysis of variance were significant for species for all ecophysiological leaf traits and there were significant differences in the AQY, LCP, LSP, Pnmax, Rd, φ, SPAD, SLA, WUEmax, and LUEmax of different woody species (*p* < 0.05) (Table 2). The maximum value of the apparent quantum efficiency (AQY) was 3.03 times the minimum value, 3.99 times for the light compensation point (LCP), 2.45 times for the light saturation point (LSP), 8.37 times for the net photosynthetic rate at light saturation (Pnmax), 4.55 times for the dark respiration rate (Rd), 6.78 times for the water potential (φ), 2.00 times for the SPAD, 3.87 times for the SLA, 4.56 times for the maximum water-use efficiency (WUEmax), and 6.90 times for the maximum light-utilization efficiency (LUEmax) (Table S1). The greater the difference between the LSP and LCP of different species, the wider the range of light suitable for growth of the species. These species also had larger Pnmax values (Figure 4).

### 2.4. Ecophysiological Traits among Different Life Forms and Families

Among different life forms, LCP, Rd, and SPAD decreased in the sequence of: evergreen shrub and shrubby bamboo > deciduous shrub and fujimoto > deciduous tree and small deciduous trees; Pnmax and LSP decreased in the sequence of: deciduous tree and small deciduous trees > deciduous shrub and fujimoto > evergreen shrub and shrubby bamboo; AQY, WUEmax, and LUEmax decreased in the sequence of: evergreen shrub and shrubby bamboo > deciduous tree and small deciduous trees > deciduous shrub and fujimoto; φ decreased in the sequence of: deciduous shrub and fujimoto > evergreen shrub and shrubby bamboo > deciduous tree and small deciduous trees; SLA decreased in the sequence of: deciduous shrub and fujimoto > deciduous tree and small deciduous trees > evergreen shrub and shrubby bamboo; the AQY, LCP, Rd, SPAD, WUEmax, and LUEmax values exhibited no significant differences among the different life forms. The Pnmax and LSP of evergreen shrubs and shrubby bamboo were significantly lower than those of deciduous trees and shrubs. The φ of deciduous tree and small deciduous trees were significantly lower than those of deciduous shrub and fujimoto, and evergreen shrub and shrubby bamboo. The SLA of deciduous shrub and fujimoto were significantly higher than those of deciduous tree and small deciduous trees, and evergreen shrub and shrubby bamboo (Table 3).

The AQY, LCP, LSP, Pnmax, Rd, φ WUEmax, and LUEmax values exhibited no significant differences among families. There were significant differences in the SLA and SPAD among families, and the SLA of Oleaceae was significantly lower than that of other families. Oleaceae had the highest SPAD, and Rosaceae had the lowest SPAD (Table 4).

### 2.5. Principal Component Analysis of 47 Woody Species Based on Ecophysiological Traits 

**Figure 5:**
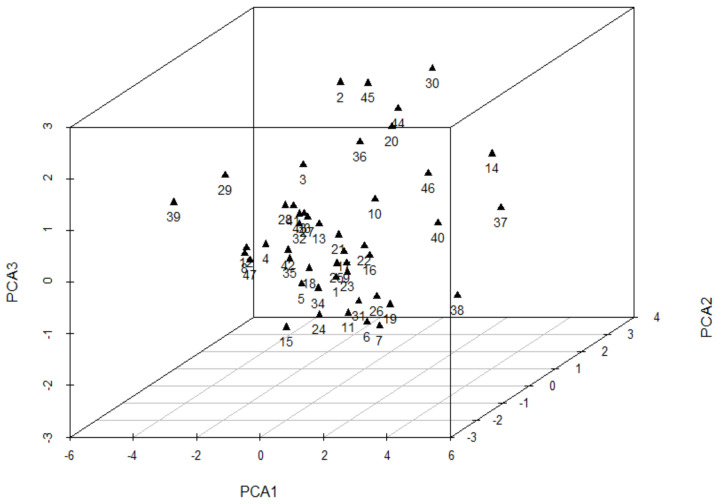
Principal component analysis diagram of 47 woody species based on ecophysiological traits. Species numbers are shown in Table 1.

Principal component analyses showed that the contribution rates of the first three principal components were 32.257%, 16.855%, and 14.544%, respectively; the cumulative contribution rate was 63.656%. Most species were distributed around the center of a three-dimensional diagram, while over ten species were scattered away from the center (Figure 5).

## 3. Discussion

The differences in the main ecophysiological indicators of the different species can further reflect the adaptability of plants to the environment [18]. A previous study showed that there are significant differences in the ecophysiological indicators of different species [21], which is consistent with our research results. In our study, the species conserved in the Beijing Botanical Garden had obvious differences in terms of the AQY, LCP, LSP, Pnmax, Rd, φ, SPAD, SLA, WUEmax, and LUEmax values, which indicated that, although these species were in similar environments for conservation, they had obvious differences in terms of ecophysiological strategies and were adapted to similar environments. The AQY of leaves is a parameter that reflects the potential of plants for the absorption, conversion, and utilization of low light [22]; the level of AQY corresponds to the efficiency of light-energy conversion by leaves [23], and it is generally no more than 0.125 μmol·μmol^−1^ [23,24]. Consistently, the AQY values of all sample species in our study were lower than this threshold; *Ginkgo biloba* L. and *Syringa pekinensis* Rupr. had a high AQY, whereas *A. truncatum* showed a low value, especially under low-light conditions. For *A. truncatum*, the AQY was 0.034 μmol·m^−2^·s^−1^, which was considerably lower than that in a natural environment, indicating a decline in light-utilization with a decrease in light availability. Rd represents the rate of respiration, or of organic matter consumption, in the dark [25]. Previous reports [26,27] showed that, under natural conditions with a high light incidence, *Ligustrum* × *vicaryi* Rehder has an Rd of 1.44 μmol·m^−2^·s^−1^. However, in our study, the Rd of *L. vicaryi* was as high as 0.94 μmol·m^−2^·s^−1^, indicating that this species, in a natural environment, consumes more photosynthates through respiration during night-time. Numerous studies have demonstrated that plants usually develop a high SLA with low light availability, and this response can help plants to increase the efficiency of photon capture and maximize carbon gains [28]. In a natural environment, a community usually appears with a high density and a multi-layered structure, and the light conditions are thus relatively insufficient in the understory [29]. Contrary to natural conditions, light availability is essentially abundant in botanical gardens as there is sparse canopy cover, resulting in a low SLA. This is consistent with our results; the SLA was 111.46 cm^2^·g^−1^ for *A. persica* L., and 137.22 cm^2^·g^−1^ for *Cotinus coggygria* Scop., which were all lower than those in natural environments [30].

### 3.1. Light-Energy-Utilization Efficiencies of Different Plant Leaves

The LUE of leaves was defined as the ratio of the net photosynthetic rate (Pn) of the leaf to the PPFD it absorbs; this parameter is widely used to evaluate the ability of plant leaves to use light energy [31]. Studies show that the LUE of most plant leaves increases with an increase in light intensity at a low light intensity, and when it exceeds a certain light intensity, the value decreases non-linearly with an increase in light intensity [32]. This is consistent with our study result, where the LUE of all species reached a maximum under a low PPFD, indicating that the low-light environmental conditions and multi-layer structures were beneficial in terms of increasing the LUE of plant leaves. The light-utilization efficiency differed among different species [33]. Generally, plants located at the top or with sufficient light had a smaller LUE, and plants located in a multi-layer structure had a larger LUE [34]. In our study, there was no significant difference in the LUEmax values of leaves of species in different life forms in the botanical garden, which may have been due to the sparse planting density of each species and less to do with the multi-layer structure due to artificial planting management. Species were grown in places with sufficient light, and they had acclimated to similar environments, resulting in no significant difference in light-utilization efficiency for different life forms in the botanical gardens.

### 3.2. Water-Use Efficiencies of Different Plant Leaves

WUE is an important ecophysiological trait and a comprehensive indicator for evaluating the degree of acclimation to water shortage [35]. In general, plants have a higher WUE under drought conditions, and have conservative water-use strategies to maintain plant growth and development; furthermore, under adequate water-environmental conditions, a lower WUE can cause plants to obtain higher productive forces [36,37]. In a forest system, water-use efficiency is the key link between tree production and water management [38]. Understanding the water-use efficiency of plants can not only help to understand the survival and adaptation strategies of plants, but it can also aide in artificially regulating limited water resources to obtain the highest yield or economic benefit [38,39]. Studies have shown that there are significant differences in the water-use efficiencies of different tree species [40,41]. However, in our study, there was no significant difference in WUE among tree species. When the PPFD value was less than 400 μmol·m^−2^·s^−1^, the WUE increased with the increasing of the PPFD; when the light intensity was greater than 400 μmol·m^−2^·s^−1^, the WUE of the species reached saturation and the value was basically the same, and was mostly concentrated around 5 μmol mmol^−1^. This may have been due to artificial watering and irrigation in the botanical garden environment, and fact that the plants had been in an environment with sufficient water for a long time, and thus, adapted to the sufficient-water environment.

### 3.3. Comparison of Ecophysiological Plant Traits for the Different Life Forms and Families

The difference in ecophysiological leaf traits among different plant groups shows the possible differences in the resource utilization of different species, and this difference will help species to make full use of environmental resources, thereby improving the stability of the entire system; the plant functional traits vary among the life forms of plants [42,43]. After plants are conserved ex situ in a botanical garden, plants will continuously adjust their physiological processes to adapt to the artificially cultivated and managed habitat and finally respond in terms of ecophysiology [44]. A study on plant ecology acknowledged that different plant species inhabiting the same environment often display similarities in terms of life form and ecophysiological traits [15]. In our study, the AQY, LCP, Rd, SPAD, WUEmax, and LUEmax values had no significant differences among the different life forms, while the LSP, Pnmax, φ, and SLA exhibited significant differences among the different life forms, indicating that species of different life forms grown in similar artificially managed environments in a botanical garden displayed no significant differences in terms of some ecophysiological traits. There were no significant differences in terms of photosynthetic indicators among the families; however, the SLA and SPAD of plant leaves were significantly different, indicating that different woody species in the artificial environment of the botanical garden acclimated to similar environments, and that the environment had a greater impact on the photosynthetic parameters, but had little effect on the SLA and SPAD of plant leaves.

## 4. Materials and Methods

### 4.1. Study Area and Experimental Materials

All test species were grown at the Beijing Botanical Garden, Institute of Botany, Chinese Academy of Sciences. The garden is located southeast of Fragrant Hill and 18 km from the center of Beijing (39°48′ N, 116°28′ E) at an elevation of 76 m above sea level. The garden has a temperate terrestrial climate, with high temperatures and rain in the summer, cold and dry winters, and short springs and autumns. The mean annual temperature is 11.6 °C, the relative humidity is 43–79%, and the mean annual precipitation is 634.2 mm [45]. The average temperature in January is −3.2 °C, the average temperature in July is 26.5 °C, the extreme minimum temperature in January is −20.2 °C, and the extreme maximum temperature in July is 41.7 °C (http://data.cma.cn/data, accesed on 20 August 2021).

Forty-seven commonly used garden woody species were selected for measurements, including twenty-six trees and twenty-one shrubs and fujimoto, comprising twenty-two families and forty-two genera in Northern China (Table 1). For each species, five individuals with good growth, no obvious diseases or insect pests, and of a consistent age were randomly selected. The selected plants were planted in the same period and in similar environments under the same management practices, based on the records of the Beijing Botanical Garden. Primary measurements occurred on sunny days during July and August of 2019. From each plant, leaves were selected from the top of the middle branches on the sunny side for in situ measurements. Only one species was measured in one day to ensure that the measurement periods were similar, and the measurement times were from 8:00 to 12:00 a.m. and from 2:00 to 4:00 p.m. Five individuals for each species, and three leaves for each individual, were measured. The soil moistures of 47 species were measured at the same time. The results of analysis of variance showed that there was no significant difference in the soil water content and soil water potential of the 47 species, and their average values in the 0–30 cm soil layer were 8.99% and −0.95 MPa, respectively.

### 4.2. Experimental Design

#### 4.2.1. Light-Response Curves 

The light-response curves (LRCs) of leaves of different species were determined with a portable photosynthesis system (Li-6400XT, LI-COR, Lincoln, NE, USA). We kept the angle and direction of the leaf’s natural attachment unchanged during measurements. A CO_2_ small steel cylinder housed a CO_2_ injection system that provided a reactive CO_2_ substrate for photosynthesis; the CO_2_ supply concentration was controlled at 400 μmol·m^−2^·s^−1^. The flow rate of the instrument was set to 500 mol·s^−1^. The temperature was controlled at about 25 °C, and the relative humidity was controlled at about 60–70%. The used light source was a red-blue LED light source with a Li-6400XT configuration, and the leaf chamber was a standard leaf chamber (2 × 3 cm^2^); thirteen differing photosynthetic photon flux densities were used at 1800, 1500, 1200, 1000, 800, 600, 400, 200, 150, 100, 50, 20, and 0 μmol·m^−2^·s^−1^, and we set the data collection time for each different light intensity value to 3–5 min [46]. We measured the net photosynthetic rate (Pn) under different PPFD values. Before measurement of the light-response curves of the photosynthetic characteristics, plant leaves were illuminated for 20–30 min at a saturated light intensity.

#### 4.2.2. Leaf Water Potential Measurement

The dawn leaf water potential (φ, MPa) was measured using a dew point water potential meter (WP4C, METER Group, Inc., NE Hopkins Court Pullmn, USA). This water potential is the most stable and highest water state of a plant in a day, and is less affected by environmental changes [47]. The WP4 instrument was warmed up for 30 min before measurements. Leaves were collected between 5:00 and 6:00 in the morning of 30 and 31 July 2019. The leaves were shredded and placed in a sample box for the WP4. The sample volume did not exceed 1/2 of the sample box volume, and the water potential value was then recorded. Leaf samples for the water potential measurements were sampled at similar positions with leaf light-response curve measurements.

#### 4.2.3. Specific Leaf Area

The leaf area (LA) was measured using the grid method, and the SLA was measured using the drying method [48]. The shape of the leaf was drawn on graph paper, and the area of the graph paper was calculated to obtain the LA. The fresh leaves were weighed and put in an envelope for transportation back to the laboratory. After drying in an oven at 105 °C for one hour, temperature was lowered to 80 °C and the leaves were dried to a constant weight; then, the dried leaves were weighed. The SLA was calculated using the following equation [49]:

SLA = LA/W (1)

where W represents the leaf dry mass.

#### 4.2.4. Relative Chlorophyll Content

Immediately after gas exchange measurements, the relative chlorophyll content of leaves in similar positions, where the light-response curves were measured, was measured using a chlorophyll meter (SPAD-502, Konica Minolta Sensing, Inc. Osaka, Japan). Three SPAD readings were taken and averaged around the leaf edge for each leaf. 

### 4.3. Data Analyses

#### 4.3.1. Light-Response Curve Fitting

The right-angle hyperbola correction model with the highest fitting accuracy was used to fit the photosynthetic response curve [50]:

(2) $$\mathrm{Pn}=\alpha \frac{1-\beta I}{1+\gamma I}I-\mathrm{Rd}$$

where α is the initial slope of the light-response curve, i.e., the AQY (μmol·m^−2^·s^−1^); β and γ are the suppression coefficient and saturation coefficient, respectively; *I* is the PPFD (μmol·m^−2^·s^−1^); and Rd is the dark respiration rate (μmol·m^−2^·s^−1^).

Using Equation (2), Pnmax, LSP, and LCP could be obtained:

(3) $$\mathrm{LSP}=\frac{\sqrt{\frac{\beta +\gamma }{\beta }}-1}{\gamma }$$

(4) $$\mathrm{LCP}=\frac{\alpha -\gamma \cdot \mathrm{Rd}-\sqrt{(\gamma \cdot \mathrm{Rd}-\alpha {)}^{2}-4\cdot \alpha \cdot \beta \cdot \mathrm{Rd}}}{2\cdot \alpha \cdot \beta }$$

(5) $$\mathrm{Pnmax}=\alpha \cdot (\frac{\sqrt{\beta +\gamma }-\sqrt{\beta }}{\gamma }{)}^{2}-\mathrm{Rd}$$

#### 4.3.2. Light-Energy-Utilization Efficiency and Water-Use Efficiency Curve Fitting

The LUE of the plant leaves could be obtained using the following equation [31,50]

(6) $$\mathrm{LUE}=\frac{\mathrm{Pn}}{I}=\alpha \frac{1-\beta I}{1+\gamma I}-\frac{\mathrm{Rd}}{I}$$

Using Equation (8), the maximum light-energy-utilization efficiency and saturated light intensity corresponding to the maximum light-energy-utilization efficiency (Il-sat) could be obtained:

(7) $$\mathrm{Il}-\mathrm{sat}=\frac{1}{\sqrt{\frac{\alpha (\beta +\gamma )}{\mathrm{Rd}}}-\gamma }$$

(8) $$\mathrm{LUEmax}=\alpha \frac{1-\beta \mathrm{Il}\text{-}\mathrm{sat}}{1+\gamma \mathrm{Il}\text{-}\mathrm{sat}}-\frac{\mathrm{Rd}}{\mathrm{Il}\text{-}\mathrm{sat}}$$

#### 4.3.3. Water-Use Efficiency

The WUE was calculated using the following equation [50,51]:

(9) $$\mathrm{WUE}=\frac{\mathrm{Pn}}{\mathrm{Tr}}=\frac{1}{\mathrm{Tr}}(\alpha \frac{1-\beta I}{1-\gamma I}I-\mathrm{Rd})=\alpha_{1}\frac{1-\beta_{1}I}{1-\gamma_{1}I}I-{\mathrm{Rd}}_{1}$$

where Rd_1_ = Rd/Tr, Tr is the transpiration rate.

From Equation (11), the maximum water-use efficiency could be obtained:

(10) $$\mathrm{Iw}\text{-}\mathrm{sat}=\frac{\sqrt{(\beta_{1}+\gamma_{1})/\beta_{1}}-1}{\gamma_{1}}$$

(11) $$\mathrm{WUEmax}=\alpha_{1}{\left(\frac{\sqrt{\beta_{1}+\gamma_{1}}-\sqrt{\beta_{1}}}{\gamma_{1}}\right)}^{2}-{\mathrm{Rd}}_{1}$$

### 4.4. Statistical Analysis

The total sample size for the light-response curves was *n* = 235 (5 replicate plants per species × 47 species). Log transform was applied to all data before statistical analyses to ensure homogeneity of variance. One-way analysis of variance was used to compare the differences in ecophysiological leaf traits for the 47 species. Differences in the ecophysiological leaf traits between life forms and between families were revealed using one-way analysis of variance using mean values of the species. All statistical analyses were performed using SPSS Statistics 21.0 (SPSS, Inc., Chicago, IL, USA). Principal component analysis (PCA) was conducted based on 10 ecophysiological traits from 47 species [52], and the results of the PCA were plotted as a graph using R 4.0.2 for Windows 4.5. 

## 5. Conclusions

Species conserved in similar environments in a botanical garden showed obvious differences in terms of ecophysiological traits. The light-saturation point, net photosynthetic rate at light saturation, φ, and SLA had significant differences among different plant life forms, while the AQY, LCP, Rd, SPAD, WUEmax, and LUEmax values showed no significant differences. There were no significant differences in photosynthetic traits among families; however, the SLA and SPAD of plant leaves were significantly different. Most of the photosynthetic traits were different at the species level (low level), while no photosynthetic traits were different at the family level (high level). The results can help us better understand the ecological adaptation strategies of plants and provide a certain theoretical basis for satisfying the growth and development conditions of plants to the greatest extent, thus improving the management level of artificial conservation in botanical gardens.

## Figures and Tables

**Table 1:** Forty-seven commonly used species in green spaces in North China.

Species (Abbreviation)	Life Forms	Family	Natural Growing Environment
*Eucommia ulmoides* (Euc ulm)	Deciduous tree	Eucommiaceae	Heliophyte, grown in valleys or low-slope sparse forests
*Ginkgo biloba* (Gin bil)	Deciduous tree	Ginkgoaceae	Heliophyte, grown in natural forest
*Magnolia biondii* (Mag bio)	Deciduous tree	Magnoliaceae	Heliophyte, grown in forest
*Liriodendron chinense* (Lir chi)	Deciduous tree	Magnoliaceae	Heliophyte, grown in mountain forests
*Acer truncatum* (Ace tru)	Deciduous tree	Aceraceae	Heliophyte (understory in mixed forests)
*Euonymus maackii* (Euo maa)	Deciduous tree	Celastraceae	Heliophyte
*Amygdalus davidiana* (Amy dav)	Deciduous tree	Rosaceae	Understorey naturally, grown on hillsides, valley bottoms
*Gymnocladus chinensis* (Gym chi)	Deciduous tree	Leguminosae	Heliophyte, grown on hillsides, mountainsides, and woods
*Sophora japonica* (Sop jap)	Deciduous tree	Leguminosae	Heliophyte, slightly shade tolerant
*Juglans regia* (Jug reg)	Deciduous tree	Juglandaceae	Heliophyte, grown on both sides of mountain valleys
*Quercus aliena* var. acutiserrata (Que ali)	Deciduous tree	Fagaceae	Heliophyte, grown in mountain woods
*Cornus officinalis* (Cor off)	Deciduous tree	Cornaceae	Heliophyte, grown on forest margins or in forests
*Kalopanax septemlobus* (Kal sep)	Deciduous tree	Araliaceae	Heliophyte, mostly found in forests, bushes and forest margins
*Diospyros kaki* (Dio kak)	Deciduous tree	Ebenaceae	Heliophyte, grown in mountains, flats or sandy beaches
*Pteroceltis tatarinowii* (Pte tat)	Deciduous tree	Ulmaceae	Heliophyte, grown in limestone mountain sparse forests along valley streams
Malus × micromalus (Mal mic)	Small deciduous tree	Rosaceae	Heliophyte
*Prunus cerasifera* ‘Atropurpurea’ (Pru cer)	Small deciduous tree	Rosaceae	Heliophyte
*Prunus blireana* ‘Meiren’ (Pru bli)	Small deciduous tree	Rosaceae	Heliophyte
*Amygdalus persica* (Amy per)	Small deciduous tree	Rosaceae	Heliophyte
*Syringa pekinensis* (Syr pek)	Small deciduous tree	Oleaceae	Heliophyte, grown in hillside shrubs, sparse forest
*Fontanesia fortunei* (Fon for)	Small deciduous tree	Oleaceae	Heliophyte, slightly shade tolerant, grown in ditches, streams, or forests
*Xanthoceras sorbifolium* (Xan sor)	Small deciduous tree	Sapindaceae	Heliophyte, slightly shade tolerant, grown on hills and slopes
*Rhamnus utilis* (Rha uti)	Small deciduous tree	Rhamnaceae	Understorey naturally, grown in mountains, hills, hillside grass, thickets or sparse forests
*Ulmus lamellose* (Ulm lam)	Small deciduous tree	Ulmaceae	Medium light-loving, grown in valleys or hillside weeds
*Chionanthus retusus* (Chi ret)	Small deciduous tree	Oleaceae	Heliophyte, grown in sparse mixed forests or thickets
*Cotinus coggygria* (Coy cog)	Small deciduous tree	Anacardiaceae	Heliophyte
*Amygdalus triloba* (Amy tri)	Deciduous shrub	Rosaceae	Heliophyte, slightly shade tolerant, grown in low to mid-altitude slopes
*Sorbaria kirilowii* (Sor kir)	Deciduous shrub	Rosaceae	Neutral species, grown in sunny hillsides and in woods
*Rhodotypos scandens* (Rho sca)	Deciduous shrub	Rosaceae	Heliophyte, grown in sparse forests on hillsides
*Forsythia suspensa* (For sus)	Deciduous shrub	Oleaceae	Heliophyte, slightly shade tolerant, grown in hillside shrubs, under forest
Ligustrum × vicaryi (Lig vic)	Deciduous shrub	Oleaceae	Heliophyte
*Kolkwitzia amabilis* (Kol ama)	Deciduous shrub	Caprifoliaceae	Heliophyte, grown on hillsides, roadsides and bushes
*Weigela florida* (Wei flo)	Deciduous shrub	Caprifoliaceae	Heliophyte, slightly shade tolerant, grown in moist valleys, shade
*Lonicera maackii* (Lon maa)	Deciduous shrub	Caprifoliaceae	Heliophyte, grown in bushes in forests
*Viburnum macrocephalum Fort.* f. keteleeri (Carrière) Rehder (Vib mac)	Deciduous shrub	Caprifoliaceae	Heliophyte, slightly shade tolerant, grown in hills, hillside forests or thickets
*Viburnum farreri* (Vib far)	Deciduous shrub	Caprifoliaceae	Heliophyte, grown in valley forests
*Lagerstroemia indica* (Lag ind)	Deciduous shrub	Lythraceae	Heliophyte, slightly shade tolerant
*Cercis chinensis* (Cer chi)	Deciduous shrub	Leguminosae	Heliophyte, slightly shade tolerant
*Kerria japonica* (Ker jap)	Deciduous shrub	Rosaceae	Heliophyte, slightly shade tolerant
*Hibiscus syriacus* (Hib syr)	Deciduous shrub	Malvaceae	Heliophyte, slightly shade tolerant
*Sambucus williamsii* (Sam wil)	Deciduous shrub	Caprifoliaceae	Heliophyte, slightly shade tolerant, grown under forest, bushes
*Celastrus orbiculatus* (Cel orb)	Deciduous shrub	Celastraceae	Heliophyte, slightly shade tolerant
*Wisteria floribunda* (Wis flo)	Deciduous fujimoto	Leguminosae	Heliophyte
*Euonymus japonicus* (Euo jap)	Evergreen shrub	Celastraceae	Heliophyte, slightly shade tolerant
*Euonymus kiautschovicus (Euo kia)*	Evergreen shrub	Celastraceae	Grown on flat ground, hillside
*Rosa chinensis* (Ros chi)	Evergreen shrub	Rosaceae	Heliophyte
*Phyllostachys propinqua* (Phy pro)	Evergreen shrubby bamboo	Poaceae	Grown in warm and humid climates

**Table 2:** One-way ANOVA of leaf ecophysiological traits of 47 species. F-values are shown, *p* < 0.05 for all F values.

Ecophysiological Traits	F
Apparent quantum efficiency (AQY)	4.87
Light compensation point (LCP)	2.05
Light saturation point (LSP)	3.00
Net photosynthetic rate at light saturation (Pnmax)	6.95
Dark respiration rate (Rd)	4.42
Water potential (φ)	11.77
Relative chlorophyll content (SPAD)	26.74
Specific leaf area (SLA)	11.09
Water-use efficiency (WUEmax)	3.62
Light-utilization efficiency (LUEmax)	3.89

**Table 3:** Results of a one-way ANOVA of ecophysiological traits of species in different life forms. The values were ecophysiological indicators for three life forms (mean ± SE), different lowercase letters indicate significant differences between different life forms for the same parameter (*p* < 0.05). n represents the number of woody species. Abbreviations are shown in Table 1.

Ecophysiological Traits	Deciduous Tree and Small Deciduous Trees (*n* = 26)	Deciduous Shrub and Fujimoto (*n* = 17)	Evergreen Shrub and Shrubby Bamboo (*n* = 4)
AQY	0.0681 ± 0.002 a	0.0679 ± 0.002 a	0.072 ± 0.004 a
LCP	19.68 ± 0.90 a	19.79 ± 1.27 a	22.11 ± 3.50 a
LSP	1217.11 ± 30.72 a	1121.75 ± 38.20 a	942.56 ± 7321 b
Pnmax	10.32 ± 0.36 a	9.88 ± 0.52 a	7.46 ± 0.56 b
Rd	1.15 ± 0.04 a	1.20 ± 0.07 a	1.30 ± 0.16 a
φ	−1.32 ± 0.03 b	−1.08 ± 0.04 a	−1.11 ± 0.05 a
SPAD	47.15 ± 0.61 a	47.99 ± 0.905 a	54.03 ± 3.05 a
SLA	141.26 ± 3.67 b	176.95 ± 9.56 a	135.32 ± 8.41 b
WUEmax	5.08 ± 0.20 a	4.51 ± 0.18 a	5.20 ± 0.49 a
LUEmax	0.029 ± 0.001 a	0.027 ± 0.001 a	0.037 ± 0.005 a

**Table 4:** Results of a one-way ANOVA of ecophysiological traits of species of different families. The values were ecophysiological indicators of five families (mean ± SE), different lowercase letters indicate significant differences among different families for the same parameter (*p* < 0.05). Abbreviations are shown in Table 1.

Ecophysiological Traits	Oleaceae (*n* = 5)	Rosaceae (*n* = 10)	Caprifoliaceae (*n* = 6)	Leguminosae (*n* = 4)	Celastraceae (*n* = 4)
AQY	0.072 ± 0.004 a	0.067 ± 0.002 a	0.067 ± 0.004 a	0.068 ± 0.004 a	0.075 ± 0.003 a
LCP	19.86 ± 1.07 a	20.99 ± 1.75 a	17.47 ± 1.17 a	22.64 ± 4.50 a	18.88 ± 1.36 a
LSP	1121.27 ± 80.08 a	1096.98 ± 51.89 a	1139.15 ± 51.29 a	1277.27 ± 73.68 a	1104.08 ± 88.53 a
Pnmax	9.92 ± 0.70 a	9.71 ± 0.54 a	8.50 ± 0.45 a	10.65 ± 1.21 a	9.58 ± 0.76 a
Rd	1.30 ± 0.10 a	1.22 ± 0.07 a	1.02 ± 0.08 a	1.32 ± 0.18 a	1.25 ± 0.10 a
φ	−1.36 ± 0.08 a	−1.28 ± 0.04 a	−1.17 ± 0.06 a	−1.17 ± 0.08 a	−1.20 ± 0.07 a
SPAD	58.07 ± 1.15 a	43.79 ± 0.77 d	48.18 ± 1.21 bc	44.62 ± 1.07 cd	54.76 ± 2.58 ab
SLA	122.87 ± 5.73 b	173.40 ± 12.54 a	166.24 ± 12.52 a	184.89 ± 14.49 a	148.56 ± 17.95 ab
WUEmax	4.87 ± 0.38 a	4.85 ± 0.27 a	4.29 ± 0.28 a	4.56 ± 0.60 a	4.78 ± 0.26 a
LUEmax	0.029 ± 0.003 a	0.028 ± 0.002 a	0.028 ± 0.002 a	0.023 ± 0.003 a	0.028 ± 0.002 a

## Data Availability

No new data were created or analyzed in this study.

## Supplementary Materials

The following supporting information can be downloaded at: https://www.mdpi.com/article/10.3390/plants11060725/s1, Table S1: Ecophysiological leaf parameters of 47 species.
