# Supplementary material for: Ecophysiological Leaf Traits of Forty-Seven Woody Species under Long-Term Acclimation in a Botanical Garden
Source: Plants (Basel). 2022 Mar 9;11(6):725. doi: 10.3390/plants11060725 (PMC8954897; doi:10.3390/plants11060725)
Supplement: Supplementary file 1 [file plants-11-00725-s001.zip › plants-1588191-supplementary.pdf]

plants

MDPI

Supplementary Material

Table S1. Ecophysiological leaf parameters of 47 species (Mean±SE, n=5). Abbreviations: apparent quantum efficiency (AQY), light compensation point (LCP), light saturation point (LSP), net photosynthetic rate at light saturation (Pnmax), dark respiration rate (Rd), leaf water potential (φ), relative chlorophyll content (SPAD), maximum water-use efficiency (WUEmax), and specific leaf area (SLA), maximum light energy use efficiency (LUEmax).

Number	Species	AQE	LCP	LSP	Pnmax	Rd	φ	SPAD	SLA	WUEmax	LUEmax
1	*Eucommia ulmoides* (Euc ulm)	0.073±0.002	16.52±2.76	1459.93±17.04	12.03±0.72	1.13±0.19	-1.27±0.16	46.31±1.52	151.27±9.13	3.97±0.29	0.024±0.003
2	*Ginkgo biloba* (Gin bil)	0.103±0.008	12.26±0.89	949.61±111.79	5.58±1.06	1.06±0.07	-1.05±0.06	52.57±2.94	115.27±8.66	4.57±0.69	0.034±0.003
3	*Magnolia biondii* (Mag bio)	0.065±0.02	28.38±12.47	944.48±207.91	2.49±0.76	0.9±0.26	-0.84±0.17	42.27±1.52	86.48±0.74	2.1±0.2	0.015±0.002
4	*Liriodendron chinense* (Lir chi)	0.072±0.004	14.27±1.7	991.86±81.56	8.73±0.91	0.93±0.09	-1.52±0.09	37.24±2.45	170.98±9.89	4.55±0.55	0.031±0.003
5	*Acer truncatum* (Ace tru)	0.034±0.011	19.62±4.9	984.06±71.88	7.1±0.67	0.49±0.09	-1.24±0.06	39.46±1.7	149.71±2.77	9.58±0.79	0.069±0.006
6	*Euonymus maackii* (Euo maa)	0.068±0.003	20.77±1.12	1436.24±127.38	13.68±0.54	1.33±0.11	-1.68±0.13	47.37±1.67	116.74±10.08	5.35±0.2	0.022±0.001
7	*Amygdalus davidiana* (Amy dav)	0.061±0.005	26.7±5.87	1435.59±64.41	15.39±1.48	1.44±0.22	-1.19±0.11	43.49±0.86	132.09±8.7	4.18±0.57	0.013±0.002
8	*Gymnocladus chinensis* (Gym chi)	0.066±0.006	12.55±2.03	1018.23±178.31	6.08±1.11	0.76±0.15	-1.65±0.09	40.66±1.7	210.72±4.59	6.47±1.87	0.034±0.007
9	*Sophora japonica* (Sop jap)	0.072±0.003	17.12±1.55	1339.08±124.27	13.85±1.02	1.15±0.08	-1.13±0.13	47.97±1.22	153.89±5.14	3.68±0.33	0.017±0.001
10	*Juglans regia* (Jug reg)	0.071±0.001	17.55±1.29	1064.55±115.47	11.47±1.27	1.15±0.07	-0.92±0.17	48.36±1.04	157.85±10.36	7.78±0.77	0.041±0.006
11	*Quercus aliena* var. *acutiserrata* (Que ali)	0.047±0.006	19.64±3.49	1283.03±87.01	13.58±1.58	0.91±0.19	-1.22±0.02	46.03±0.54	182.59±11.59	7.79±1.93	0.05±0.019
12	*Cornus officinalis* (Cor off)	0.065±0.003	11.17±1.89	1144.21±367.14	6.23±1.05	0.67±0.14	-1.48±0.06	43.01±0.55	165.4±4.04	3.73±0.48	0.033±0.004
13	*Kalopanax septemlobus* (Kal sep)	0.064±0.008	14.33±4.17	1134.16±28.43	9.35±1.15	0.85±0.23	-1.00±0.13	44.29±1.11	138.74±10.8	5.37±0.73	0.041±0.004
14	*Diospyros kaki* (Dio kak)	0.084±0.004	23.07±1.78	1554.09±76.38	13.81±0.89	1.74±0.12	-0.27±0.08	64.23±2.72	105.65±5.22	4.8±0.31	0.02±0.002

plants

15	*Pteroceltis tatarinowii* (Pte tat)	0.054±0.004	19.84±2.24	1562.36±159.22	8.2±0.89	0.95±0.09	-1.53±0.08	44.6±1.02	184.5±7.3	3.59±0.32	0.03±0.003
16	Malus × micromalus (Mal mic)	0.077±0.004	17.63±1.25	1313.54±135.97	12.27±1.01	1.26±0.11	-1.54±0.07	51.36±1	117.75±19.13	5.44±0.84	0.028±0.004
17	*Prunus cerasifera* 'Atropurpurea' (Pru cer)	0.065±0.004	27.39±4.38	1098.89±61.43	9.91±0.78	1.54±0.16	-1.21±0.07	39±1.1	149.39±7.82	4.48±0.48	0.021±0.004
18	*Prunus blireana* 'Meiren' (Pru bli)	0.072±0.009	18.03±3.61	1318.27±144.44	9.11±0.81	1.08±0.13	-1.47±0.06	41.69±1.59	165.89±18.57	4.95±0.42	0.033±0.004
19	*Amygdalus persica* (Amy per)	0.069±0.003	21.21±2.37	1376.19±182.81	13.97±1.49	1.37±0.15	-1.53±0.22	45.49±1.28	111.46±2.81	6.98±1.27	0.03±0.005
20	*Syringa pekinensis* (Syr pek)	0.102±0.005	21.11±2.94	1012.86±221.43	7.93±1.75	1.77±0.21	-1.58±0.05	52.77±1.24	128.03±8.98	5.78±0.84	0.036±0.007
21	*Fontanesia fortunei* (Fon for)	0.064±0.006	14.98±2.1	1171.48±133.06	11.95±1.42	0.93±0.2	-1.32±0.06	62.43±1.21	147.42±6.72	3.83±0.47	0.019±0.004
22	*Xanthoceras sorbifolium* (Xan sor)	0.066±0.006	18.76±2.98	1285.03±125.46	10.96±2.28	1.1±0.14	-1.14±0.16	47.67±1.34	124.07±24.01	5.97±0.82	0.035±0.006
23	*Rhamnus utilis* (Rha uti)	0.082±0.002	16.24±1.41	1217.09±124.7	15.27±0.68	1.27±0.11	-1.63±0.06	47.56±0.53	144.97±16.38	4.41±0.4	0.02±0.002
24	*Ulmus lamellosa* (Ulm lam)	0.05±0.007	25.42±2.82	1266.68±112.21	7.82±1.23	1.18±0.23	-1.83±0.02	49.28±1.02	98.79±13.86	3.68±0.83	0.023±0.005
25	*Chionanthus retusus* (Chi ret)	0.066±0.005	21.42±1.52	1125.63±185.1	10.62±0.99	1.29±0.07	-1.75±0.1	53.64±1.97	125.9±11.58	3.9±0.42	0.02±0.002
26	*Cotinus coggygria* (Cot cog)	0.053±0.005	35.91±11.66	1157.7±182.47	10.86±2.43	1.59±0.42	-1.52±0.08	47.21±1.25	137.22±6.07	5.06±0.88	0.02±0.004
27	*Amygdalus triloba* (Amy tri)	0.068±0.004	15.84±2.67	1030.47±100.5	10.29±0.83	0.98±0.13	-0.87±0.18	38.64±0.75	169.11±22.75	4.95±0.34	0.028±0.003
28	*Sorbaria kirilowii* (Sor kir)	0.06±0.006	26.74±3.22	717.41±49.4	6.75±0.97	1.35±0.07	-1.4±0.02	42.24±0.57	217.22±11.07	4.61±0.45	0.027±0.003
29	*Rhodotypos scandens* (Rho sca)	0.058±0.003	10.47±0.11	638.64±15.21	5.95±0.3	0.57±0.02	-1.15±0.06	46.75±1.69	197.86±38.95	3.65±0.34	0.032±0.002
30	*Forsythia suspensa* (For sus)	0.076±0.004	23.78±1.91	800.5±136.45	7.14±1.18	1.57±0.17	-0.63±0.09	60.87±3.44	113.2±4.57	7.07±0.86	0.045±0.009

plants

MDPI

31	Ligustrum × vicaryi (Lig vic)	0.055±0.003	18±1.87	1495.88±91.67	11.96±1.45	0.94±0.13	-1.51±0.02	60.62±0.97	99.82±15.35	3.75±0.42	0.027±0.003
32	*Kolkwitzia amabilis* (Kol ama)	0.049±0.003	19±1.19	905.18±46.23	8.15±0.76	0.88±0.08	-0.79±0.17	39.76±1.02	121.52±7.82	4.16±0.83	0.033±0.004
33	*Weigela florida* (Wei flo)	0.082±0.002	13.18±1.44	1310.26±144.29	8.83±1.37	0.98±0.09	-1.11±0.09	41.63±0.43	211.77±21.38	5.45±0.81	0.039±0.007
34	*Lonicera maackii* (Lon maa)	0.052±0.004	20.42±2.47	1163.48±172.66	10.39±0.77	0.98±0.1	-1.69±0.04	57.76±1.57	137.77±4.62	3.82±0.45	0.018±0.002
35	*Viburnum macrocephalum* Fort. f. *keteleeri* (Carrière) Rehder (Vib mac)	0.049±0.005	11.94±2.48	1091.88±95.7	8.79±0.84	0.5±0.04	-1.2±0.06	50.96±1.71	115.59±5.72	4.67±0.74	0.033±0.003
36	*Viburnum farreri* (Vib far)	0.089±0.008	23.36±3.91	1170.23±110.75	6.37±0.94	1.63±0.23	-1.14±0.06	51.69±0.35	183.72±5.63	4.18±0.69	0.027±0.005
37	*Lagerstroemia indica* (Lag ind)	0.081±0.005	22.91±2.1	1251.69±147.71	20.83±2.01	1.8±0.24	-0.68±0.06	58.15±2.53	110.25±9.36	6.77±0.28	0.03±0.003
38	*Cercis chinensis* (Cer chi)	0.062±0.012	41.75±15.68	1556.23±98.12	14.35±3.23	2.23±0.53	-1.02±0.05	48.63±1.83	134.65±28.49	3.38±0.68	0.01±0.003
39	*Kerria japonica* (Ker jap)	0.069±0.006	12.46±1.54	771.86±36.02	5.9±0.36	0.79±0.12	-1.38±0.05	36.96±0.41	334.79±13.7	3.35±0.37	0.029±0.007
40	*Hibiscus syriacus* (Hib syr)	0.08±0.006	27.75±3.71	1442.32±130.72	14.92±2.19	2.03±0.31	-0.77±0.04	50.9±0.56	146.65±11.14	3.33±0.51	0.012±0.001
41	*Sambucus williamsii* (Sam wil)	0.076±0.007	16.92±2.26	1193.85±132.41	8.45±1.44	1.12±0.11	-1.09±0.01	47.28±0.48	227.08±40.74	3.45±0.43	0.02±0.002
42	*Celastrus orbiculatus* (Cel orb)	0.072±0.002	12.72±2.19	1334.38±162.57	10.63±0.96	0.86±0.14	-1.01±0.03	41.83±1.79	246.85±12.2	5.36±0.52	0.033±0.005
43	*Wisteria floribunda* (Wis flo)	0.07±0.007	19.12±2.35	1195.54±86.21	8.32±1.54	1.15±0.11	-0.9±0.09	41.2±0.69	240.31±12.12	4.72±0.85	0.031±0.006
44	*Euonymus japonicus* (Euo jap)	0.071±0.005	23.59±1.55	819.64±110.74	7.66±0.61	1.46±0.08	-1.04±0.07	67.85±1.97	101.05±7.35	4.05±0.41	0.025±0.004
45	*Euonymus kiautschovicus* (Euo kia)	0.087±0.007	18.46±3.3	826.07±116.59	6.36±1.23	1.36±0.29	-1.07±0.07	61.98±2.38	129.6±11.65	4.36±0.68	0.031±0.006
46	*Rosa chinensis* (Ros chi)	0.077±0.005	33.38±12.63	1268.91±198.74	7.52±1.67	1.8±0.41	-1.03±0.02	52.34±0.49	138.4±7.13	5.95±1.6	0.036±0.014
47	*Phyllostachys propinqua* (Phy pro)	0.051±0.008	13.01±1.99	855.63±28.27	8.31±0.85	0.58±0.08	-1.3±0.15	33.96±1.35	172.24±5.16	6.44±0.58	0.053±0.009
